# MiR-221 and miR-222 target PUMA to induce cell survival in glioblastoma

**DOI:** 10.1186/1476-4598-9-229

**Published:** 2010-09-02

**Authors:** Chun-Zhi Zhang, Jun-Xia Zhang, An-Ling Zhang, Zhen-Dong Shi, Lei Han, Zhi-Fan Jia, Wei-Dong Yang, Guang-Xiu Wang, Tao Jiang, Yong-Ping You, Pei-Yu Pu, Jin-Quan Cheng, Chun-Sheng Kang

**Affiliations:** 1 Department of Neurosurgery, Tianjin Medical University General Hospital, Tianjin 300052, China; 2 Laboratory of Neuro-Oncology, Tianjin Medical University General Hospital, Tianjin 300052, China; 3 Department of Radiation Oncology, Tianjin Huanhu hospital, Tianjin 300060, China; 4 Tianjin Key Laboratory of Nerve Injury, Variation and Regeneration, Tianjin 300052, China; 5 Department of Neurosurgery, Tiantan Hospital, Capital Medical University, Beijing 100050 China; 6 Department of Neurosurgery, The First Affiliated Hospital of Nanjing Medical University, Nanjing 210029, PR China; 7 Departments of Molecular Oncology, H. Lee Moffitt Cancer Center and Research Institute, Tampa, FL 33612, USA

## Abstract

**Background:**

MiR-221 and miR-222 (miR-221/222) are frequently up-regulated in various types of human malignancy including glioblastoma. Recent studies have reported that miR-221/222 regulate cell growth and cell cycle progression by targeting p27 and p57. However the underlying mechanism involved in cell survival modulation of miR-221/222 remains elusive.

**Results:**

Here we showed that miR-221/222 inhibited cell apoptosis by targeting pro-apoptotic gene PUMA in human glioma cells. Enforced expression of miR-22/222 induced cell survival whereas knockdown of miR-221/222 rendered cells to apoptosis. Further, miR-221/222 reduced PUMA protein levels by targeting PUMA-3'UTR. Introducing PUMA cDNA without 3'UTR abrogated miR-221/222-induced cell survival. Notably, knockdown of miR-221/222 induces PUMA expression and cell apoptosis and considerably decreases tumor growth in xenograft model. Finally, there was an inverse relationship between PUMA and miR-221/222 expression in glioma tissues.

**Conclusion:**

To our knowledge, these data indicate for the first time that miR-221/222 directly regulate apoptosis by targeting PUMA in glioblastoma and that miR-221/222 could be potential therapeutic targets for glioblastoma intervention.

## Background

MicroRNAs (miRNAs), a new class of small RNA (~22nt), are thought to negatively regulate protein-coding genes by base-pair matching with 3'UTR of mRNA. Growing evidence has indicated that the important roles for miRNAs in the development of different cancers. Deregulation of miRNAs has been observed in various types of human malignancy, including lymphoma, colorectal cancer, lung cancer, breast cancer, papillary thyroid carcinoma, hepatocellular carcinoma and glioblastoma [1-7]. And oncomiRs and tumor suppressor miRNAs exert their functions through regulation of tumor suppressor genes and oncogenes, respectively [8-10].

Glioblastoma is one of the most common forms of neural malignancy, with a median survival of 9-12 months. Despite the significant toxicities of current therapies, a large fraction of brain cancer patients suffer tumor recurrence due to the resistance to chemo- and radio-therapy [11-13]. Thus, there are urgent needs to develop novel therapeutic approaches by targeting the molecules that are altered in this malignancy. Recent studies showed frequent deregulation of miR-221/222 in glioblastoma [1,14]. We profiled miRNA expression in five glioblastoma cell lines, one astrocytoma cell line and one normal brain tissue, and found that miR-221/222 were overexpressed with a greater than 2-fold increase in all glioma cell lines [15]. We and others have shown that miR-221/222 induce cell growth and cell cycle progression through negative regulation of p27 and p57 [16-19]. In the current study, we have demonstrated that miR-221/222 are of important role in regulation of cell apoptosis by direct targeting pro-apoptotic molecule PUMA in cell culture and xenograft model. Knockdown of miR-221/222 induces apoptosis and reduces tumor growth as well as upreulates PUMA expression. Whereas, ectopic expression of miR-221/222 exhibits opposite effects. Moreover, miR-221/222 directly interact with 3'UTR of PUMA to repress PUMA expression. These findings indicate that PUMA is a bona fide target of miR-221/222 and these 2 miRNAs could be critical therapeutic targets for glioblastoma intervention.

## Results

### Critical role of miR-221/222 in apoptosis pathway

**Figure 1:**
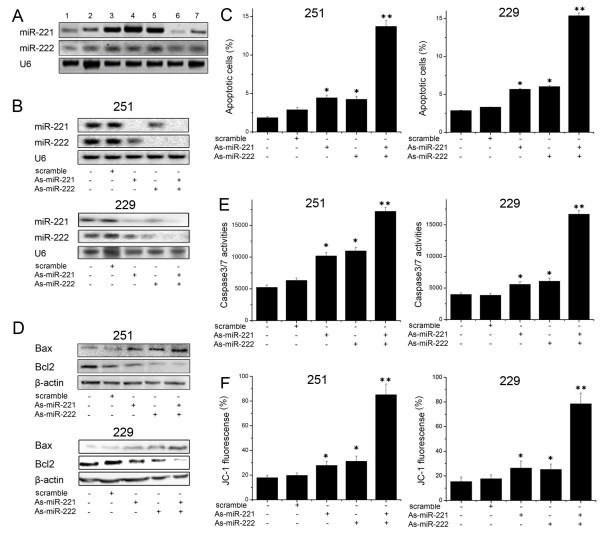
**As-miR-221/222 increases glioma apoptosis**. (A) Identification of differential expression of miR-221/222 in glioma cell lines (1, A172; 2, TJ905; 3, TJ899; 4, U251; 5, TJ866; 6, H4; 7, LN229) by Northern blot analysis. (B) U251 and LN229 cells were transfected with As-miR-221/222, and miR-221/222 expression level was detected by Northern blot assay. U6 was regarded as endogenous normalizer. (C) Annexin V analysis showed that U251 and LN229 cells transfected with As-miR-221/222 displayed significantly more apoptosis than the other four groups. (D) U251 and LN229 cells were transfected with As-miR-221/222, and Bax and Bcl2 protein level was detected by Western blot assay. β-actin protein was regarded as endogenous normalizer. (E) A significant increase in caspase 3/7 activity was detected in U251 and LN229 cells transfected with As-miR-221/222. (F) U251 cells transfected with As-miR-221/222 showed a significantly greater collapse in mitochondrial membrane potential compared with the other four groups. * P < 0.05 compared with control group, ** P < 0.01 compared with control group.

Previous studies have documented miR-221 and miR-222 regulation of cell cycle progression and cell proliferation by targeting p27 and p57 [16-18]. However, their role in apoptotic pathway has not been well studied. As initial step, we carried out Northern blot analysis of miR-221 and miR-222 expression in a panel of glioma cell lines. Fig. 1A showed that U251, TJ866 and TJ899 cells expressed higher levels of miR-221 and miR-222 compared to other cell lines. To examine biological significance of miR-221 and miR-222 in glioma, U251 and LN229 cells were treated with As-miR-221 and/or As-miR-222 (Fig. 1B). Interestingly, annexin V-labeling revealed that knockdown of miR-221 and miR-222 significantly increased cell apoptosis compared to the cells treated with scramble oligonucleotide (Fig. 1C). Moreover, Western blot assay displayed that pro-apoptotic protein Bax expression was significantly up-regulated while BCL2 expression was down-regulated in As-miR-221/222 group (Fig. 1D). In addition, caspase 3/7 activity was also considerably elevated in miR-221 and miR-222 knocked down cells (Fig. 1E). Since collapse of the mitochondrial membrane potential is one of the early events in apoptosis [20], we next examined if miR-221 and miR-222 regulate mitochondrial membrane potential. The cells with depletion of miR-221 and miR-222 were stained with cationic dye JC-1. FACSCalibur analysis showed that the mitochondrial membrane potential was largely damaged when miR-221 and miR-222 were depleted (Fig. 1F). These findings indicate that miR-221 and miR-222 play an important role in initiation of cell apoptosis.

### PUMA is a target for miR-221 and miR-222

**Figure 2:**
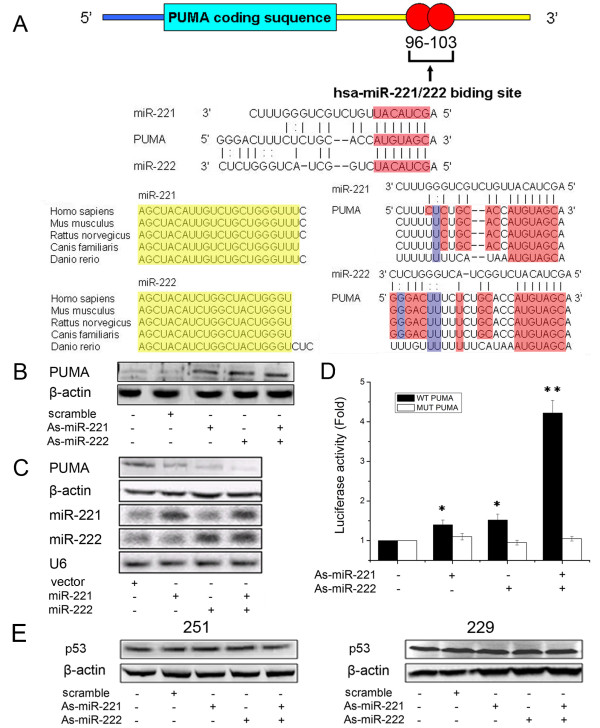
**PUMA was a direct target gene of miR-221/222**. (A) Schematic representation of the putative binding sites in PUMA mRNAs 3'UTR for miR-221/222 (identical seed sequences AGCUACAU as shown). Sequence alignment of miR-221, miR-222 and the conserved binding sites among the different vertebrate species (Red, G:C pair or A:U pair; Blue, G:U pair). (B) U251 cells were transfected with As-miR-221/222, and PUMA protein level was detected by Western blot assay. β-actin protein was regarded as endogenous normalizer. (C) H4 cells were transfected with pMSCV-miR-221/222, and miR-221/222 and PUMA expression levels were detected by Northern blot and Western blot assay. U6 and β-actin protein were regarded as endogenous normalizer. (D) pGL3-WT-PUMA-3'UTR-Luc and pGL3-MUT-PUMA-3'UTR-Luc reporters were transfected into U251 cells transfected with As-miR-221 and/or As-miR-222. Luciferase activity was determined 48 h after transfection. The ratio of normalized sensor to control luciferase activity is shown. Error bars represent standard deviation and were obtained from three independent experiments. (E) U251 and LN229 cells were transfected with As-miR-221/222, and p53 protein level was detected by Western blot assay. β-actin protein was regarded as endogenous normalizer. * P < 0.05 compared with control group, ** P < 0.01 compared with control group.

To determine the mechanism by which miR-221 and miR-222 regulate cell apoptosis, we performed miRNA target search using Pictar and found 3'UTR of PUMA containing the highly conserved putative miR-221 and miR-222 binding sites (Fig. 2A). Further, we knocked-down miR-221/222 in U251 cells, which exhibit elevated level of miR-221/222, and ectopically expressed miR-221/222 in H4 cells with low endogenous miR-221/222 expression (Fig. 1A). Western blot analysis showed that PUMA expression was up-regulated in U251 cells with knockdown of miR-221/222 (Fig. 2B), whereas downregulated in H4 cells overexpressing miR-221/222 (Fig. 2C), compared to the cell treated with scrambled oligonucleotide or vector alone. Moreover, we created pGL3-WT-PUMA-3'UTR and pGL3-MUT-PUMA-3'UTR plasmids. Reporter assay revealed reduction of miR-221/222 led to a marked increase of luciferase activity of pGL3-WT-PUMA-3'UTR plasmid, without change in luciferase activity of pGL3-MUT-PUMA-3'UTR plasmid (Fig. 2D). PUMA recently identified as a critical mediator of p53-associated apoptosis. Thus, we further explored the effect of miR-221/222 on p53 expression. There was no change of p53 in U251 and LN229 cells after reduction of miR-221/222 (Fig. 2E). These indicate that miR-221/222 directly modulate PUMA expression by binding 3'UTR of PUMA.

### Expression of PUMA overrides miR-221/222 survival function

**Figure 3:**
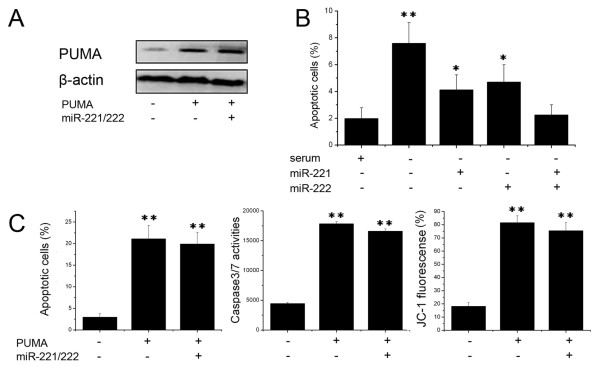
**Expression of PUMA abrogates miR-221/222 survival function**. (A) H4 cells were transfected pcDNA-PUMA (not including the 3'UTR), and PUMA expression was measured by Western blot assay. β-actin protein was regarded as endogenous normalizer. (B) H4 cells were transfected with pMSCV-miR-221/222, then treated with serum starvation and cell apoptosis was Annexin V analysis. (C) H4 cells were transfected with pcDNA-PUMA (not including the 3'UTR) and pMSCV-miR-221/222, and cell apoptosis, caspase activity and mitochondrial membrane potential were measured by Annexin V analysis, caspase 3/7 activity assay and JC-1 staining. * P < 0.05 compared with control group, ** P < 0.01 compared with control group.

Having demonstrated PUMA as a direct target of miR-221 and miR-222, we next examined the importance of PUMA in miR-221/222-mediated cell survival. Since H4 expressed low level of miR-221/222, we transfected PUMA lacking 3'UTR together with and without miR-221 and miR-222 into the H4 cells (Fig. 3A). Annexin V analysis showed that expression of miR-221 and/or miR-222 significantly reduced cell apoptosis induced by serum starvation (Fig. 3B). However, expression of PUMA largely abrogated miR-221/222 effects on cell apoptosis (Fig. 3C), suggesting that PUMA is a critical target of miR-221 and miR-222 involved in cell apoptosis.

### As-miR-221/222 inhibits glioblastoma xenograft growth accompanying PUMA upregulation

**Figure 4:**
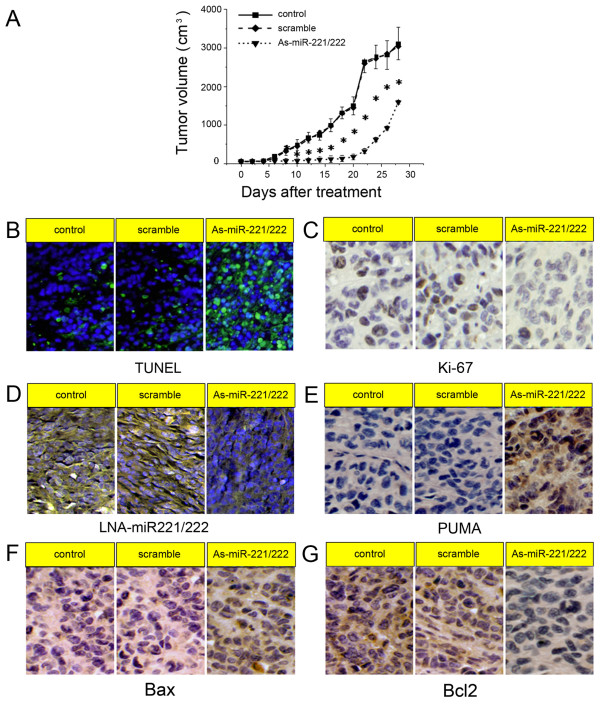
**Knock-down of miR-221/222 inhibits tumor growth in a xenograft mouse model**. (A) When subcutaneous tumors were established, As-miR-221/222 were injected in a multi-site injection manner every 3 days for 15 days. Tumor volumes were measured every 2 days during treatment. (B) TUNEL assay in xenograft tumor sections revealed that As-miR-221/222 induced cell apoptosis. (C) Ki-67 expression was detected by immunohistochemistry assay in xenograft tumor sections. (D) Fluorescence in situ hybridization showed that As-miR-221/222 effectively inhibited the expression of miR-221 and miR-222. (E-G) PUMA, Bax and Bcl2 expression was detected by immunohistochemistry assay in xenograft tumor sections. * P < 0.05 compared with control group.

Since miR-221 and miR-222 are frequently elevated in glioblastoma and play an important role in cell survival, we further examined the effects of knockdown of miR-221/222 on tumor growth. As shown in Fig. 4A, tumors continued growing in both scramble and control groups. However, As-miR-221/222 significantly reduced tumor growth (p < 0.05). After 5 days of withdraw of the treatment, tumor started to regrow. TUNEL assay analysis of xenograft tumor taken at 28 days after treatment revealed much more apoptosis in As-miR221/222 group when compared to tumors from scramble and control groups (Fig. 4B). We also histologically observed that there were more neonatal micro-vessels, bigger tumor cell nuclei, chromosomes stained a deeper blue, more mitotic tumor cells and fewer necrotic foci in control and scramble groups than those in As-miR221/222 group. Ki-67 staining shows that As-miR-221/222 treated tumors had a lower proliferation index compared with the control groups (Fig. 4C). In addition, LNA-ISH analysis confirmed that miR-221/222 levels were considerably reduced in As-miR221/222 group (Fig. 4D). Immunohistochemical stanining analysis revealed that PUMA levels were up-regulated in As-miR221/222 group (Fig. 4E), confirming the data in vitro that PUMA as a direct target of miR-221/222. Additionally, Bax expression was increased, whereas Bcl2 expression was decreased in xenograft tumor sections (Fig. 4G, F). These findings further indicate that miR-221/222 targets PUMA and that As-miR-221/222 could be therapeutic means for glioblastoma intervention.

### Inverse correlation of expression of miR-221/222 and PUMA in glioma tissues

**Figure 5:**
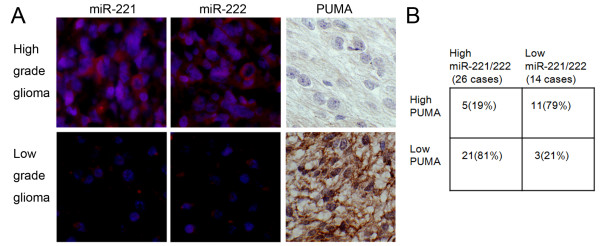
**MiR-221/222 inversely correlate with PUMA expression in glioma tissues**. (A) Expression of miR-221/222 and PUMA was analyzed in representative of gliomas with LNA-ISH and immunohistochemical staining. Expression levels of miR-221/222 and PUMA were quantified as described in methods. (B) Chisquare test analysis of miR-221/222 and PUMA expression. The inverse correlation is significant (p < 0.001).

Having demonstrated PUMA as a major target of miR-221/222, we further investigated the correlation of between miR-221/222 and PUMA expression in gliomas. We examined 40 human glioma specimens with LNA-ISH and immunohistochemical staining. Representative images of miR-221/222 and PUMA were shown in Fig. 5A. Upregulation of miR-221/222 was detected in 26 gliomas (Fig. 5B). Of the 26 tumors with elevated miR-221/222, 21 (81%) had low levels of PUMA (P < 0.001). 11 of 14 (79%) specimens with downregulated miR-221/222 presented high levels of PUMA. In addition, we found that miR-221/222 expression increased significantly in high grade gliomas compared with low grade gliomas.

## Discussion

Previous studies have shown frequent upregulation of miR-221/222 in human malignancies incuding glioblastoma [15]. MiR-221/222 regulate cell cycle through targeting p27 and p57 [16-19]. In the present study, we demonstrated the role of miR-221/222 in regulation of cell apoptosis. Knockdown of miR-221/222 induced change of mitochondrial membrane potential and caspase-mediated apoptosis. Further, we proved that pro-apoptotic protein PUMA was negatively regulated by miR-221/222. In addition, local injection of As-miR-221/222 reduced tumor growth and induced apoptosis in xenograft model. These findings provide the evidence of direct regulation of mitochondrial apoptotic pathway by miR-221/222.

A mitochondrial-dependent step in apoptosis, involving mitochondrial outer membrane permeabilization (MOMP), is associated with most pro-apoptotic stimuli. This process is controlled by both pro- and anti-apoptotic members of the Bcl2 family and leads to the release of mitochondrial apoptotic factors such as cytochrome c, Smac/DIABLO and Omi/HtrA2 into the cytosol. Pro-apoptotic protein Bax induces a selective process of MOMP through the formation of channels or pores after oligomerization, allowing the release of proteins localized within the intermembrane space such as cytochrome c, which triggers caspase 9/3 activation to induce the apoptotic phenotype [21]. However, Willis had detected no association of PUMA with Bax [22]. Other studies identified that PUMA indirectly activated Bax by binding and inactivating anti-apoptotic Bcl2 family members that include Bcl2 protein [23,24]. In our study, we found that knockdown miR-221/222 could downregulate Bcl2 and upregulate Bax by western blot assay. In addition, the change of BAX and Bcl2 expression in xenograft study with U251 cells confirmed the data in vitro. These results indicate that miR-221/222 negatively regulate PUMA which leads to decrease Bcl2 and increase BAX.

Our results proved that modulating effect of miR-221/222 on PUMA by directly targeting PUMA. Bioinformatics analysis showed that 3'UTR of PUMA mRNA existed the highly conserved putative miR-221/222 binding sites. Luciferase reporter assay validated that PUMA was a direct target of miR-221/222. Our xenograft study with U251 cells also shows that As-miR-221/222 treatment reduces tumor growth accompanying increase of PUMA expression and apoptosis. Further, there was an inverse relationship between PUMA and miR-221/222 expression levels in glioma tissues. PUMA, as a key mediator of p53-associated apoptosis, actually accounts for nearly all of the apoptotic activity attributed to p53. However, the expression level of p53 remained unchanged after reduction of miR-221/222. Thus, these finding suggest that PUMA is a core target of miR-221/222 in cell apoptosis.

## Conclusions

In summary, our data demonstrated that miR-221/222 induced cell survival by direct targeting PUMA, and thus regulates mitochondrial pathway. We also provided direct evidence using As-miR-221/222 as therapeutic approaches for glioblastoma. The role of miR-221/222 in gilomagensis needs to be further investigated by creating transgenic mouse model.

## Methods

### Cell culture and transfection

Human glioblastoma cells (A172, U251, H4, LN229), retrovirus-packaging cells PT67 and the mouse fibroblast cell line NIH3T3 were obtained from the China Academia Sinica Cell Repository, Shanghai, China. Human glioblastoma cells (TJ905, TJ899, TJ866) were established in our lab. The cells were maintained in Dulbecco's modified Eagle's medium (DMEM) (Gibco) supplemented with 10% fetal bovine serum (Gibco), 2 mM glutamine (Sigma), 100 μg/ml penicillin (Sigma), and 100 μg/ml streptomycin (Sigma), and incubated at 37°C with 5% CO_2_. Cell transfection were used Lipofectamine 2000 (Invitrogen) following manufacture instruction.

### Plasmids, virus production and oligonucleotides

Human pMSCV-miR-221 and -miR-222 were kindly provided by. R. Agami (The Netherlands Cancer Institute, Amsterdam, The Netherlands). The retroviruses expressing miR-221 or miR-222 were obtained by transfection of pMSCV-miR-221 and -miR-222 into PT67 packaging cells and selected with blasticidin S (10 μg/ml) for six weeks. The viruses were tittered in NIH3T3 cells. Once cells grew to 60% confluence, cells were infected with pMSCV-miR-221 and/or pMSCV-miR-222 at a multiplicity of infection (MOI) of 50. HA-tagged wild-type PUMA in pCDNA3 was obtained from B. Volgelstein [25]. pGL3-WT-PUMA-3'UTR-Luc reporter was created by ligation of PCR products of 3'UTR of PUMA into the XbaI site of the pGL3 control vector (Promega, USA). The primers for PCR amplification are: PUMA-3'UTR-Forward: 5'-TCA TGA ATTC GCC CCT CCC ACC TCC TGA CAC CCT GGC CAG CGC GGG GGA CTT TCT CTG C and PUMA-3'UTR-Reverse: 5'-CGC CCC CGG GAC AGG CAG GGC TGG GAG TCC AGT ATG CTA CAT GGT GCA GAG AAA GTC CC-3'. pGL3-MUT-PUMA-3'UTR-Luc reporter was generated from pGL3-WT-PUMA-3'UTR-Luc reporter by deleting the binding site for miR-221/222. The 2'-OMe-oligonucleotides were chemically synthesized and purified by high-performance liquid chromatography by GenePharma Co., Ltd. (Shanghai, China). The sequences are: 2'-OMe-As-miR-221 (As-miR-221), 5'-AGCUACAUUGUCUGCUGGGUUUC-3'; 2'-OMe-As-miR-222 (As-miR-222), 5'-AGCUACAUCUGGCUACUGGGU-3'. 200 pmol As-miR-221 and/or As-miR-222 were transfected using Lipofectamine 2000 (Invitrogen). Cells transfected with scrambled 2'-OMe oligonucleotides (scramble) were used as control.

### RNA extraction and Northern blot analysis

Total RNA was isolated from cells using TRIzol reagent (Invitrogen) as previously described [26]. For Northern blotting, total RNAs (20 μg) were separated on 12% denaturing polyacrylamide gels, and then transferred to Hybond N+ nylon membrane (Ambion). Following the UV cross-linked, he membrane was hybridized with digoxigenin (DIG)-labeled miR-221 and miR-222 probes overnight in a buffer containing 5× SSC, 20 mmol/L Na_2_HPO_4 _(pH 7.2), 7% SDS, 1× Denhardt's solution and 0.2 mg/mL salmon sperm DNA. The membrane was washed with 1× SSC/1% SDS at 50°C. After equilibration in detection buffer, blots were detected with a DIG Luminescent Detection Kit (Roche, USA) and analyzed by GeneGenius.

### Western blot, miRNA locked nucleic acid (LNA) *in situ *hybridization, immunohistochemistry and luciferase reporter assay

Western blot, miRNA-LNA in situ hybridization and immunohistochemistry were performed as previously described [27]. MiR-221 and miR-222-LNA oligonucleotides contained locked nucleic acids at five locations (underlined): 5'-GAA ACC CAG CAG ACA ATG TAG CT-3' (miR-221); 5'-GAG ACC CAG TAG CCA GAT GTA GCT-3' (miR-222). For reporter assay, cells were cultured in 96-well plates and transfected with pGL3-PUMA-3'UTR-Luc, and As-miR-221 and/or As-miR-221. Following 48 h incubation, luciferase activity was measured using a dual-luciferase reporter system (Promega).

### Apoptosis

48 h after transfection, apoptosis in cultured cells was evaluated with annexin V labeling, caspase 3/7 activity and mitochondrial membrane potential. For the annexin V assay, an annexin V-FITC labeled Apoptosis Detection Kit (Abcam) was used according to the manufacturer's protocol. Caspase 3/7 activity was measured using Caspase-Glo 3/7 reagent (Promega). Mitochondrial membrane potential was determined with cationic dye JC-1 (5,5',6,6'-tetrachloro-1,1',3,3'- tetraethylbenzimidazolylcarbocyanine-chloride/C25H27Cl3N4) staining [21]. Briefly cells were harvested and first stained with PI. Following wash twice with PBS, cells were incubated with 10 mg/ml JC-1 for 20 min at room temperature and then analyzed with FACSCalibur to detect green fluorescence at excitation/emission wavelengths of 485/530 nm and red fluorescence at excitation/emission wavelengths of 485/590 nm. TUNEL assay was used to detect the apoptosis in tumor specimens and was performed as previously described [15].

### Nude Mouse Tumor Xenograft Model **and As-miR-221/222 treatment**

U251 glioma cells were subcutaneously injected to 5-week old female nude mice (Cancer Institute of The Chinese Academy of Medical Science). When the tumor volume reached 50 mm^3^, the mice were randomly divided into three groups (ten mice per group) which were treated with 200 pmol scramble oligo, As-miR-221 and As-miR-222 in 10 μl Lipofectamine or PBS through local injection of xenograft tumor in multiple sites. The treatment was performed once every 3 days for 15 days. The tumor volume was measured with a caliper every 2 days, using the formula: volume = length × width^2 ^/2.

### Patients and samples

From 40 glioma patients, we obtained 40 primary tumor samples during surgical resection of the lesion. Immediately after surgery, samples were snap-frozen and stored in liquid nitrogen. Histological diagnosis and grading of tumors were carried out with WHO criteria (World Health Organization, 2007). A total of 18 tumors were classified as low-grade gliomas, 10 as anaplastic astrocytomas, and the remaining 12 as glioblastoma multiformes (GBMs). Then miRNA-LNA in situ hybridization and immunohistochemistry were performed. Sections with no labeling or with fewer than 5% labeled cells were scored as 0. Sections were scored as a 1 with labeling of 5-30% of cells, as a 2 with 31-70% of cells and as a 3 with labeling of ≥71%. The staining intensity was scored similarly, with 0 used for negative staining, 1 for weakly positive, 2 for moderately positive and 3 for strongly positive. The scores for the percentage of positive tumor cells and for the staining intensity were added to generate an immunoreactive score for each specimen. The product of the quantity and intensity scores were calculated such that a final score of 0-1 indicated negative expression (-), 2-3 indicated weak expression (+), 4-5 indicated moderate expression (++) and 6 indicated strong expression (+++). Each sample was examined separately and scored by two pathologists.

### Statistical Analysis

Statistical evaluation for data analysis was determined by t test. Differences with P < 0.05 were considered statistically significant

## Competing interests

The authors declare that they have no competing interests.

## Authors' contributions

CZZ, ALZ and ZDS performed the experimental work. JXZ interpreted the data and helped to draft the manuscript. LH, ZFJ, GXW and TJ participated in the experiments. WDY and YPY analyzed data. PYP, JQC and CSK conceived of the study and participated in its design and coordination. All authors have read and approved the final manuscript.
